# Optimization of the Maximum Skin Dose Measurement Technique Using Digital Imaging and Communication in Medicine—Radiation Dose Structured Report Data for Patients Undergoing Cerebral Angiography

**DOI:** 10.3390/diagnostics11010014

**Published:** 2020-12-23

**Authors:** Koichi Morota, Takashi Moritake, Keisuke Nagamoto, Satoru Matsuzaki, Koichi Nakagami, Lue Sun, Naoki Kunugita

**Affiliations:** 1 Department of Radiology, Shinkomonji Hospital, 2-5 Dairishinmachi, Moji-ku, Kitakyushu, Fukuoka 800-0057, Japan; morota@shinkomonji-hp.jp (K.M.); qqey494d@adagio.ocn.ne.jp (S.M.); 2 Department of Radiobiology and Hygiene Management, Institute of Industrial Ecological Sciences, University of Occupational and Environmental Health, Japan, 1-1 Iseigaoka, Yahatanishi-ku, Kitakyushu, Fukuoka 807-8555, Japan; k-nagamoto0201@clnc.uoeh-u.ac.jp (K.N.); nakagami@clnc.uoeh-u.ac.jp (K.N.); 3 Department of Radiology, Hospital of the University of Occupational and Environmental Health, Japan, 1-1 Iseigaoka, Yahatanishi-ku, Kitakyushu, Fukuoka 807-8556, Japan; 4 Health and Medical Research Institute, Department of Life Science and Biotechnology, National Institute of Advanced Industrial Science and Technology (AIST), Central 6, 1-1-1 Higashi, Tsukuba, Ibaraki 305-8566, Japan; lue.sun@aist.go.jp; 5 Department of Occupational and Community Health Nursing, School of Health Sciences, University of Occupational and Environmental Health, Japan, 1-1 Iseigaoka, Yahatanishi-ku, Kitakyushu, Fukuoka 807-8555, Japan; kunugita@med.uoeh-u.ac.jp

**Keywords:** DICOM-RDSR, maximum skin dose, air kerma at the patient entrance reference point, radio-photoluminescence glass dosimeter, cerebral angiography, neurointerventional radiology

## Abstract

Understanding the maximum skin dose is important for avoiding tissue reactions in cerebral angiography. In this study, we devised a method for using digital imaging and communication in medicine—radiation dose structured report (DICOM-RDSR) data to accurately estimate the maximum skin dose from the total air kerma at the patient entrance reference point (Total K_a,r_). Using a test data set (*n* = 50), we defined the mean ratio of the maximum skin dose obtained from measurements with radio-photoluminescence glass dosimeters (RPLGDs) to the Total K_a,r_ as the conversion factor, CF_Ka,constant_, and compared the accuracy of the estimated maximum skin dose obtained from multiplying Total K_a,r_ by CF_Ka,constant_ (Estimation Model 1) with that of the estimated maximum skin dose obtained from multiplying Total K_a,r_ by the functional conversion factor CF_Ka,function_ (Estimation Model 2). Estimation Model 2, which uses the quadratic function for the ratio of the fluoroscopy K_a,r_ to the Total K_a,r_ (K_a,r_ ratio), provided an estimated maximum skin dose closer to that obtained from direct measurements with RPLGDs than compared with that determined using Estimation Model 1. The same results were obtained for the validation data set (*n* = 50). It was suggested the quadratic function for the K_a,r_ ratio provides a more accurate estimate of the maximum skin dose in real time.

## 1. Introduction

The advances in interventional radiology (IVR) technology in recent years have resulted in an increased number of patients undergoing lengthy procedures, and the increased radiation exposure of patients is becoming a great concern. Although neurointerventional radiology (NIR) has a number of practical benefits for patients, including being less physically invasive than surgical treatment and requiring a shorter time in hospital, there have been numerous reported cases of tissue reactions (deterministic effects), such as hair loss, under increased radiation exposure doses [1,2,3,4,5].

The most important factor in managing patient radiation exposure dose is to ascertain the maximum skin dose (D_skin,max_) during an IVR procedure [6], and assessing the D_skin,max_ in real time is important to avoid damaging the patient’s skin [7]. The reported methods for measuring doses in real time include measuring the dose at the crystalline lens of the eye using a metal–oxide–semiconductor field-effect transistor (MOSFET) dosimeter [8] and attaching up to four photoluminescence sensors (of non-toxic phosphor) to the patient’s back to measure the skin dose [9]. Because both methods show up on X-ray fluoroscopy and involve a limited number of measurement sensors, these sensors must not only be placed at locations where they do not interfere with X-ray fluoroscopy but also require the location of the maximum dose to be predicted in advance. Both methods also involve wired measurements, and their value for estimating D_skin,max_ is limited. Wireless dosimeter systems utilizing plastic scintillators have recently been developed [8,10,11]. Because these systems are not made of metal, they do not show up using X-ray fluoroscopy, and their absence of cables increases their convenience. If the number of points that can be measured simultaneously were increased, this method would become more widespread [10]. A method of mapping the exposure dose on a diagram of the human body using the biplane dose tracking system (Biplane-DTS), developed by the angiography device manufacturer Canon Medical Systems, Inc. (formerly Toshiba Medical Systems, Co.), was also reported [12]. However, although it is extremely useful to be able to visualize the dose in real time, the device-specific nature of this system means that it can only be used in limited situations. A method for estimating the dose on the patient’s body surface by performing a real-time Monte Carlo simulation using a high-speed graphics processing unit (GPU) has also been reported [13], but this system can also only currently be used in a specific facility.

**Scheme 1:**
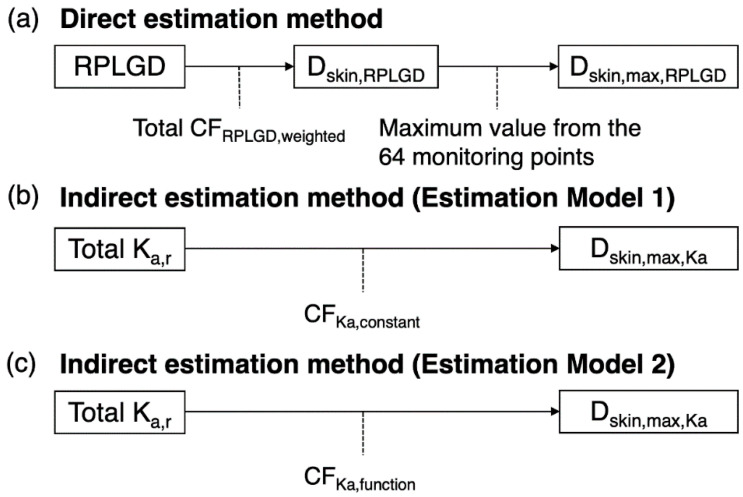
Schema of the methods used for estimating the maximum skin dose (D_skin,max_) in neurointerventional radiology (NIR) patients. (**a**) Direct estimation method using radio-photoluminescence glass dosimeters (RPLGDs) in the RADIREC^®^ system (D_skin,max,RPLGD_). Methods for indirectly estimating the maximum skin dose from the Total K_a,r_ displayed by the angiography system (D_skin,max,Ka_): (**b**) Estimation Model 1, applying a constant as the conversion factor (CF_Ka,constant_) and (**c**) Estimation Model 2, applying an individual conversion factor obtained by means of a function (CF_Ka,function_).

When considering alternative means of direct D_skin, max_ measurements in real time, the correlation between the indirect measurement value, the air kerma at the patient entrance reference point (K_a,r_) displayed on the device, and the directly measured D_skin, max_ has been widely discussed [14,15,16,17,18,19,20]. For directly measuring D_skin,max_, methods using thermoluminescent dosemeters (TLDs) [14,15,20] and Gafchromic film [15,16,17,18,19,20] have been reported. It was shown that the correlation between K_a,r_ and the directly measured D_skin,max_ is high, and if the K_a,r_ values are measured accurately, D_skin,max_ can be sufficiently predicted by indirect measurement through K_a,r_. We previously estimated the D_skin,max_ for patients undergoing NIR using a RADIREC^®^ system (Chiyoda Technol Corporation, Tokyo, Japan) (Scheme 1a) [3,21,22,23,24,25]. However, the greatest disadvantages of using the RADIREC system, a passive dosimeter system with 64 radio-photoluminescence glass dosimeters (RPLGDs) (GD-302M, Chiyoda Technol Corporation, Tokyo, Japan), for measuring the dose in a single patient are the time and effort required to obtain readouts from the RPLGDs, which make it impracticable to ascertain the D_skin,max_ in real time during NIR and to take measures at the right time to avoid damaging patient skin. As an alternative method, we analyzed the D_skin,max_ measured by the RADIREC system (D_skin,max,RPLGD_) and the total air kerma at the patient entrance reference point (Total K_a,r_) in the same patient among an appropriate number of patients (approximately 50). We used the ratio of the D_skin,max,RPLGD_ and the Total K_a,r_ (D_skin,max,RPLGD_/Total K_a,r_) as the Total K_a,r_ to the D_skin,max,Ka_ conversion factor (CF_Ka,constant_) (Scheme 1b) [21,22,23,24,25]. Using this CF_Ka,constant_ enabled the D_skin,max_ (D_skin,max,Ka_) to be estimated from the Total K_a,r_ in real time during procedures, thereby enabling the operator to be warned when the dose approaches the thresholds for skin reddening and hair loss at 2 and 3 Gy, respectively.

The Total K_a,r_ value must be recorded in a Digital Imaging and Communication in Medicine—Radiation Dose Structured Report (DICOM-RDSR) (Table 1) [26]. As this value is constantly displayed on the angiography monitor during procedures, setting the CF_Ka,constant_ before commencing the procedure would make any subsequent estimates of the D_skin,max,Ka_ in real time simpler and more convenient. However, because the irradiation angle during the procedure varies markedly between patients, the value of D_skin,max_/Total K_a,r_ also varies markedly, and converting Total K_a,r_ to D_skin,max,Ka_ using a single fixed value (CF_Ka,constant_), as in Estimation Model 1 (Scheme 1b), naturally generates large errors. Therefore, we developed a technique to convert D_skin,max,Ka_ by applying a conversion factor corrected in real time using DICOM-RDSR data for the individual patient concerned (CF_Ka,function_), as in Estimation Model 2 (Scheme 1c).

In this study, to optimize the process for estimating D_skin,max,Ka_ to establish a method that would bring the value of D_skin,max,Ka_ estimated indirectly from the Total K_a,r_ value closer to the more directly estimated D_skin,max,RPLGD_ value, we first analyzed the factors giving rise to variation in the D_skin,max,RPLGD_/Total K_a,r_ ratio and then devised a new method for correcting for this variation. Finally, we validated the efficacy of this new correction method using a separately prepared validation data set. Our objective was to improve the accuracy with which the D_skin,max_ for patients undergoing NIR can be estimated from the Total K_a,r_ to help prevent skin damage by providing the operator with real-time D_skin,max_ measurements during NIR procedures. This method may provide a new means of utilizing DICOM-RDSR data.

## 2. Materials and Methods

### 2.1. Data Sets

The test data set comprised 50 patients who underwent cerebral angiography in our hospital between October 2015 and July 2016 (diagnostic cerebral angiography: 43 cases; NIR: 7 cases), and the validation data set comprised 50 patients who underwent cerebral angiography in our hospital between August 2016 and September 2017 (diagnostic cerebral angiography: 43 cases; NIR: 7 cases) (Table 2).

### 2.2. X-ray Equipment

Angiography was performed using a single-plane angiography system (BRANSIST Safire VC9 Slender, Shimadzu Co., Kyoto, Japan) equipped with a flat-panel detector. The tube voltage and tube current were adjusted via auto exposure control, and scanning was conducted at a fluoroscopy pulse rate of 15 pulses/s and an exposure frame rate of 3 frames/s. A 1.5 mm Al + 0.6 mm Cu filter was automatically selected and applied during fluoroscopy, and a 1.0 mm Al filter was applied during exposure.

### 2.3. Dosimetry of Skin Dose for Patients Who Undergo NIR Procedures

The skin dose (D_skin,RPLGD_) from the patient’s head to their neck was measured using the RADIREC^®^ system [3,21,22,23,24,25]. This system consists of 64 RPLGDs (GD-302M, Chiyoda Technol, Corporation, Tokyo, Japan), which are passive dosimeters, placed on a special cap that covers the entire circumference of the head. The maximum skin dose (D_skin,max,RPLGD_) can thus be obtained from the dose distribution and measurements at these 64 points [24,25].

### 2.4. RPLGD X-Ray Energy Calibration

**Figure 1:**
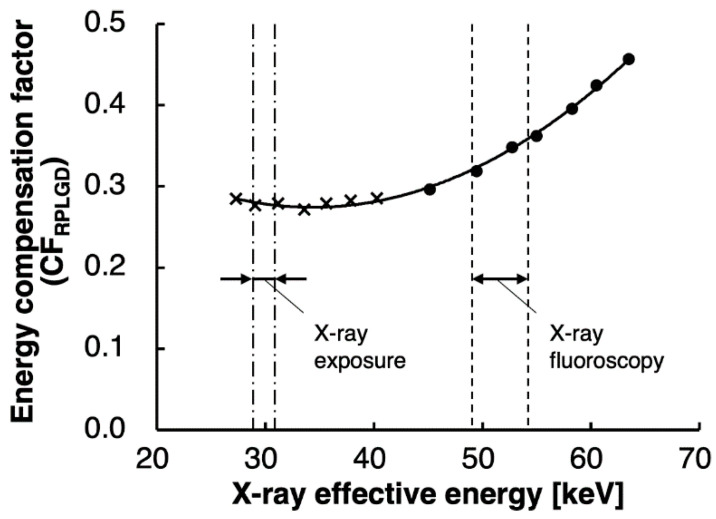
Relationship between X-ray effective energy and the radio-photoluminescence glass dosimeter (RPLGD) energy compensation factor (CF_RPLGD_). The symbols ● and × show the data obtained under simultaneous X-ray exposure to the ionization chamber and RPLGDs under settings of fluoroscopy and exposure, respectively. CF_RPLGD_ (*y*) was fitted to the following quadratic equation for X-ray effective energy (*x*): *y* = 0.0002*x*^2^ − 0.0147*x* + 0.5270 (R = 0.999) (solid line).

Firstly, to obtain the energy responses of the RPLGDs under the fluoroscopy settings, the X-ray tube voltage was increased from 60 to 120 kVp in 10 kVp increments, and the X-ray effective energy at each tube voltage was measured using the aluminum half-value layer method [27] with an ionization chamber dosimeter (AE-1322 exposure ratemeter, Applied Engineering Inc., Kiyose, Tokyo, Japan), which is calibrated annually by the Japan Quality Assurance Organization (JQA), Japan’s secondary standard body. Secondly, under the same fluoroscopy settings, the ionization chamber and the five RPLGDs were simultaneously exposed to X-rays in free air at tube voltage values from 60 to 120 kVp in 10 kVp increments. Thirdly, under the exposure settings, simultaneous irradiation of the ionization chamber and the five RPLGDs was performed using the same method as described above. Finally, the RPLGD energy compensation factors (CF_RPLGD_) for the X-ray effective energies were calculated by dividing the ionization chamber dosimeter measurements by the RPLGD readings, and the CF_RPLGD_ (*y*) values were fitted to a quadratic equation (Equation (1)) for the X-ray effective energy [keV] (*x*) (Figure 1):

*y* = 0.0002*x*^2^ − 0.0147*x* + 0.5270 (1)

### 2.5. Direct Estimation Method: Estimation of D_skin,max,RPLGD_ from RPLGD Measurements

In the cerebral angiography of actual patients, the tube voltage changes constantly in response to factors including the objective and procedure, scanning site, and patient’s position. Hence, the X-ray effective energy is also constantly changing. For this reason, we first calculated the CF_RPLGD_ from Equation (1) using the representative effective energies for fluoroscopy and the exposure obtained from the individual DICOM-RDSR data for the 50 patients in the test data set; then, we calculated the weighted calibration factor (CF_RPLGD,weighted_) from the fluoroscopy K_a,r_ and the exposure K_a,r_. We next defined the Total CF_RPLGD,weighted_ as the mean CF_RPLGD,weighted_ for all 50 patients and converted the RPLGD readout values to D_skin,RPLGD_ according to Equation (2) below (Scheme 1a):

D_skin,RPLGD_ = Total CF_RPLGD,weighted_ × RPLGD readout value (2)

While using the RADIREC system, we assumed that the maximum value of all D_skin,RPLGD_ values at the 64 dose monitoring points were D_skin,max,RPLGD_.

### 2.6. Indirect Estimation Method: Estimation of D_skin,max_ from Total K_a,r_ by Applying an Arbitrary Constant as a Conversion Factor (CF_Ka,const_) (Estimation Model 1)

The mean value of the ratio between D_skin,max,RPLGD_ and Total K_a,r_ (D_skin,max,RPLGD_/Total K_a,r_) for the 50 patients in the test data set was defined as CF_Ka,constant_, and D_skin,max,Ka_ was estimated using Equation (3) below (Scheme 1b):

D_skin,max,Ka_ = CF_Ka,constant_ × Total K_a,r_ (3)

### 2.7. Indirect Estimation Method: Estimation of D_skin,max_ from Total K_a,r_ by Applying an Arbitrary Function as a Conversion Factor (CF_Ka,function_) (Estimation Model 2)

We analyzed the associations between the D_skin,max,RPLGD_/Total K_a,r_ and the Total K_a,r_, Fluoroscopy K_a,r_, Exposure K_a,r_, Fluoroscopy Time, Number of DSA, Number of Frames, and the Fluoroscopy K_a,r_/Total K_a,r_ (K_a,r_ ratio) in the various combinations from the DICOM-RDSR data recorded for the 50 patients in the test data set. In light of the results, we used the Total K_a,r_ to D_skin,max,Ka_ conversion factor (CF_Ka,function_), an arbitrary function that minimizes the error between the estimated D_skin,max,Ka_ and the D_skin,max,RPLGD_, to estimate the D_skin,max,Ka_ for each individual patient according to Equation (4) (Scheme 1c):

D_skin,max,Ka_ = CF_Ka,function_ × Total K_a,r_ (4)

### 2.8. Comparison of the Accuracy of the Estimation of D_skin,max,Ka_ under Estimation Models 1 and 2

Using the 50 patient test data set, we carried out a regression analysis between the values of D_skin,max,Ka_ estimated indirectly using the two maximum skin dose estimation models above (Estimation Models 1 and 2) and the value of D_skin,max,RPLGD_ estimated directly from RPLGD readouts. We calculated the root mean squared error (RMSE), mean absolute error (MAE), and coefficient of determination (*R*^2^) between D_skin,max,Ka_ and D_skin,max,RPLGD_, and compared the goodness of fit of the two estimation models.

### 2.9. Validation of the Accuracy of Estimation Models 1 and 2 Using the Validation Data Set

Using the 50 patient validation data set, after first determining that there was little variation in CF_RPLGD,weighted_, we determined the Total CF_RPLGD,weighted_. We then compared the goodness of fit of the two maximum skin dose estimation models (Estimation Models 1 and 2) via the same method as that used for the test data set.

### 2.10. Statistical Analysis

SPSS (Version 25. SPSS Inc., Chicago, IL, USA) was used for statistical analyses. Differences between the mean values of the test data set and the validation data set were tested for significance using Welch’s *t*-test, with *p* < 0.05 regarded as indicating significance.

### 2.11. Ethical Approval

This study was approved by the Ethics Committee of Shinkomonji Hospital (Approval No. 27004, 10 June 2015).

## 3. Results

### 3.1. Direct Estimation of D_skin,max,RPLGD_

Table 3 shows the values of CF_RPLGD,weighted_, the RPLGD compensation factors weighted by the K_a,r_ for fluoroscopy, and the exposure obtained from the DICOM-RDSR data for the 50 patients in the test data set. CF_RPLGD,weighted_ exhibited little variation at 0.272 ± 0.004 (mean ± standard deviation; range: 0.267−0.284), suggesting that, in practical terms, the effect of patient differences on CF_RPLGD,weighted_ is negligible, so a value of 0.272 for Total CF_RPLGD,weighted_ was adopted. The highest of the D_skin,RPLGD_ values at the 64 sites calculated for each patient was used as D_skin,max,RPLGD_.

### 3.2. Indirect Estimation of D_skin,max,Ka_ Using Estimation Model 1

The D_skin,max,RPLGD_/Total K_a,r_ for the 50 patients in the test data set was 0.575 ± 0.075 (mean ± standard deviation; range: 0.425−0.795), so a value of 0.575 for CF_Ka,constant_ was used to estimate D_skin,max,Ka_ using Equation (3).

### 3.3. Indirect Estimation of D_skin,max,Ka_ Using Estimation Model 2

**Figure 2:**
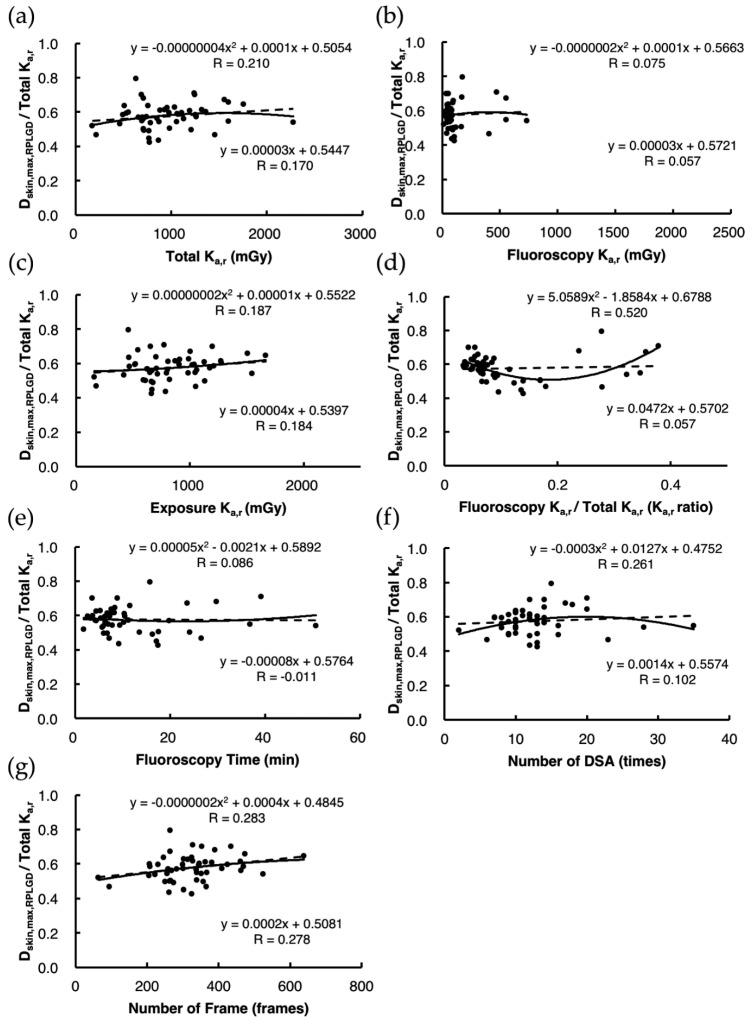
Analysis of factors affecting D_skin,max,RPLGD_ /Total K_a,r_ using the test data set (*n* = 50). The broken lines indicate linear regression, and the solid lines indicate quadratic regression. We analyzed the correlations between D_skin,max,RPLGD_/Total K_a,r_ and the following DICOM-RDSR parameters: (**a**) Total K_a,r_; (**b**) Fluoroscopy K_a,r_; (**c**) Exposure K_a,r_; (**d**) Fluoroscopy K_a,r_/Total K_a,r_ (K_a,r_ ratio); (**e**) Fluoroscopy Time; (**f**) Number of DSA; (**g**) Number of frames.

A linear regression analysis of the 50 patients in the test data set did not show any significant correlation between Total K_a,r_, Fluoroscopy K_a,r_, Exposure K_a,r_, Fluoroscopy Time, Number of DSA, Number of Frames, or Fluoroscopy K_a,r_/Total K_a,r_ (K_a,r_ ratio) and D_skin,max,RPLGD_/Total K_a,r_ (Figure 2). However, quadratic regression analysis identified a moderate correlation (R = 0.520) for Fluoroscopy K_a,r_/Total K_a,r_ (K_a,r_ ratio) alone (Figure 2d), so Equation (5) was used as the CF_Ka,function_:

CF_Ka,function_ = 5.0589 × (Fluoroscopy K_a,r_/Total K_a,r_)^2^ − 1.8584 × (Fluoroscopy K_a,r_/Total K_a,r_) + 0.6788 (5)

D_skin,max,Ka_ was estimated using Equations (4) and (5).

### 3.4. Comparison of the Accuracy of D_skin,max,Ka_ Estimated Using Estimation Models 1 and 2

**Figure 3:**
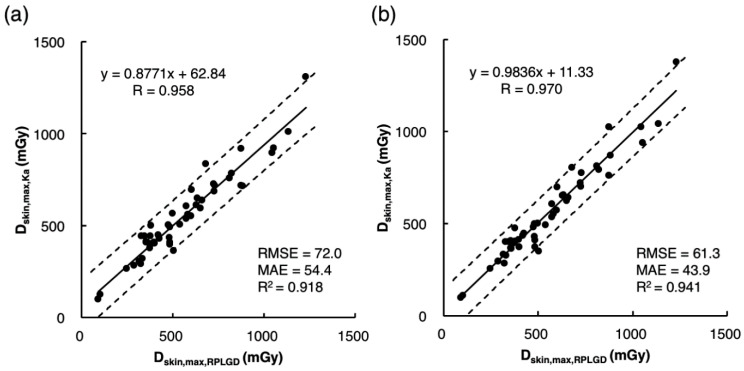
Investigation of the accuracy of the two indirect methods for estimating D_skin,max,Ka_ using the test data set (*n* = 50). (**a**) Correlation between the value of D_skin,max,Ka_ indirectly estimated using an arbitrary constant CF_Ka,constant_ (Estimation Model 1) and D_skin,max,RPLGD_ directly estimated using radio-photoluminescence glass dosimeters (RPLGDs); (**b**) correlation between the value of D_skin,max,Ka_ indirectly estimated using an arbitrary quadratic function CF_Ka,function_ (Estimation Model 2) and D_skin,max,RPLGD_ directly estimated using RPLGDs. The broken lines indicate 95% predictive intervals.

Using the 50 patient test data set, we analyzed the correlations between the values of D_skin,max,Ka_ estimated using Estimation Models 1 and 2 and D_skin,max,RPLGD_. We found that the correlation was high for both estimation methods (Model 1, R = 0.958; Model 2, R = 0.970) but that Estimation Model 2, which used CF_Ka,function_ as the conversion factor for individual patients, exhibited a better goodness of fit than Estimation Model 1 in terms of RMSE, MAE, and R^2^, demonstrating the superiority of Estimation Model 2 (Figure 3).

### 3.5. Validation of the Accuracy of Estimates Using Estimation Models 1 and 2 under the Validation Data Set

**Figure 4:**
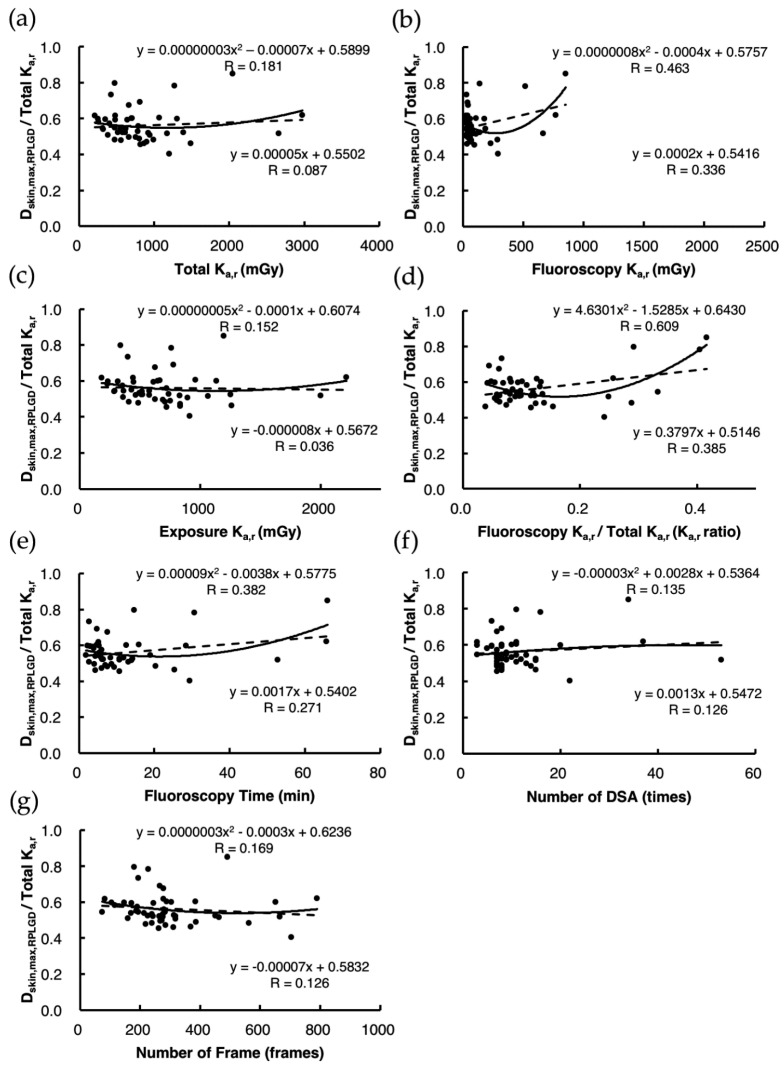
Analysis of the factors affecting D_skin,max,RPLGD_ /Total K_a,r_ using the validation data set (*n* = 50). The broken lines indicate linear regression, and the solid lines indicate quadratic regression. We analyzed the correlations between D_skin,max,RPLGD_/Total K_a,r_ and the following Digital Imaging and Communication in Medicine—Radiation Dose Structured Report parameters: (**a**) Total K_a,r_; (**b**) Fluoroscopy K_a,r_; (**c**) Exposure K_a,r_; (**d**) Fluoroscopy K_a,r_/Total K_a,r_ (K_a,r_ ratio); (**e**) Fluoroscopy Time; (**f**) Number of DSA; and (**g**) Number of frames.

In the 50 patient validation data set, CF_RPLGD,weighted_ exhibited little variation at 0.273 ± 0.004 (mean ± standard deviation; range: 0.270−0.287) (Table 4), so a value of 0.273 for Total CF_RPLGD,weighted_ was adopted. D_skin,max,RPLGD_/Total K_a,r_ was 0.562 ± 0.089 (mean ± standard deviation; range: 0.403−0.850), so a value of 0.562 was used for CF_Ka,constant_. As in the test data set, linear regression did not show any significant correlation between Total K_a,r_, Fluoroscopy K_a,r_, Exposure K_a,r_, Fluoroscopy Time, Number of DSA, Number of Frames, or Fluoroscopy K_a,r_/Total K_a,r_ (K_a,r_ ratio), or D_skin,max,RPLGD_/Total K_a,r_ (Figure 4). However, quadratic regression identified a moderate correlation (R = 0.609) for Fluoroscopy K_a,r_/Total K_a,r_ (K_a,r_ ratio) (Figure 4d), so the quadratic equation shown as Equation (6) was used as the CF_Ka,function_:

CF_Ka,function_ = 4.6301 × (Fluoroscopy K_a,r_/Total K_a,r_)^2^ − 1.5285 × (Fluoroscopy K_a,r_/Total K_a,r_) + 0.6430 (6)

**Figure 5:**
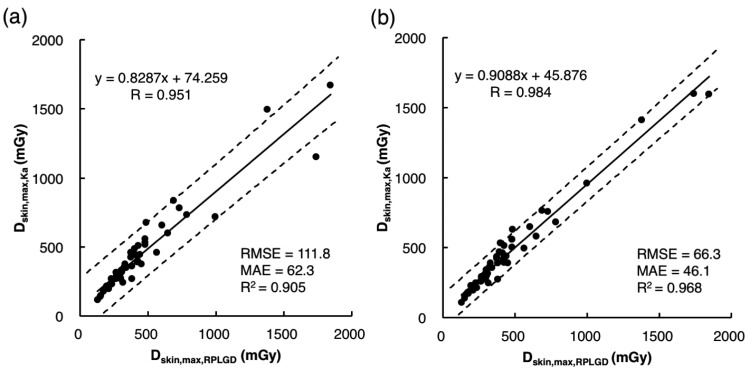
Investigation of the accuracy of the two indirect methods of estimating D_skin,max,Ka_ using the validation data set (*n* = 50). (**a**) Correlation between the value of D_skin,max,Ka_ estimated indirectly using an arbitrary constant CF_Ka,constant_ (Estimation Model 1) and D_skin,max,RPLGD_ directly estimated using RPLGDs; (**b**) correlation between the value of D_skin,max,Ka_ estimated indirectly using an arbitrary quadratic function CF_Ka,function_ (Estimation Model 2) and D_skin,max,RPLGD_ directly estimated using radio-photoluminescence glass dosimeters (RPLGDs). The broken lines indicate 95% predictive intervals.

Analysis of the correlations between the values of D_skin,max,Ka_ estimated by Estimation Models 1 and 2 using these values and D_skin,max,RPLGD_ showed that although the correlations were high for both estimation methods (Model 1, R = 0.951; Model 2, R = 0.984), Estimation Model 2, which used CF_Ka,function_ as the conversion factor for individual patients, exhibited a better goodness of fit than Estimation Model 1 in terms of the RMSE, MAE, and R^2^, demonstrating the superiority of Estimation Model 2 (Figure 5).

## 4. Discussion

Two factors are important for reducing the occurrence of radiation damage in patients undergoing IVR: minimizing stochastic effects, such as carcinogenesis and genetic effects, and avoiding tissue reactions, such as hair loss and skin injury [6].

One method for reducing the stochastic effects of IVR is to use the diagnostic reference level (DRL) to keep the radiation dose administered to the patient “as low as reasonably achievable (ALARA)” while guaranteeing the image quality required for diagnostic imaging [28,29,30]. Countries belonging to the European Union (EU) are required to establish DRLs [31], and individual countries have adopted DRLs appropriate to their situations. In the United States, organizations including the American College of Radiology (ACR), the American Association of Physicists in Medicine (AAPM), and the National Council on Radiation Protection and Measurements (NCRP) require that both image quality and dose be optimized using both the DRL, defined as the 75th percentile of the dose distribution of a number of representative facilities, and the achievable dose, defined as the 50th percentile, although not all states have adopted this approach [32]. The first Japanese DRLs were issued on 7 June 2015 by the Japan Network for Research and Information on Medical Exposure (J-RIME) [33], and these DRLs were revised five years later on 3 July 2020. With respect to NIR, the revised version includes the DRL values for the K_a,r_ and air kerma area product (P_KA_) for the imaging of six major patient groups for the three purposes of preoperative diagnostic angiography, postoperative diagnostic angiography, and endovascular treatment [34]. However, the establishment of DRLs and dose optimization by individual institutions are not directly helpful for avoiding tissue reactions. Rather, what is important is to be aware of the threshold levels in advance (reddening: 2 Gy; hair loss: 3 Gy), monitoring the D_skin,max_ in real time during NIR procedures, and informing the operator, as required, if this value approaches the threshold value [6].

A wide range of data is acquired for DICOM-RDSR, including the tube current and voltage, scanning data (such as exposure time and number of exposures), distance from the X-ray focal point to the detector, open area of the irradiation aperture, entrance angle, area dose, and patient entrance reference point dose. Because these data are acquired automatically for each fluoroscopy and exposure event, they can be used to manage medical radiation exposure for patients undergoing IVR [35,36,37,38,39,40,41], and case studies of patient dose monitoring in multiple institutions have been reported [35,36,41]. In particular, Total K_a,r_ is constantly displayed on the angiography system monitor during treatment procedures, and its recording in DICOM-RDSR is also obligatory [26], meaning that it can be used to estimate the D_skin,max_ simply and in real time at every medical facility where NIR is performed. In NIR, however, because the direction of X-ray irradiation and the extent of irradiation are constantly changing, the accurate estimation of D_skin,max_ is not necessarily simple, and the discrepancy between the values of Total K_a,r_ and D_skin,max_ mean that each individual institution should use its own conversion coefficient.

We previously analyzed D_skin,max,RPLGD_ using the RADIREC system and estimated D_skin,max,Ka_ intraoperatively in real time by multiplying the Total K_a,r_ by the mean D_skin,max,RPLGD_/Total K_a,r_ ratio as CF_Ka,constant_ (Schema 1b). However, Total K_a,r_ is the sum of all the X-ray entrance angles, and as the X-ray entrance angles are completely different for each patient, the D_skin,max,Ka_ is often larger or smaller than the actual D_skin,max,RPLGD_. Theoretically, if the X-ray entrance angle does not change at all during the procedure, the value of D_skin,max,RPLGD_/Total K_a,r_ increases and approaches 1. Conversely, if the X-ray entrance angle varies widely, the ratio will be lower. However, to our best knowledge, no index that provides an appropriate indication of variation in the X-ray entrance angle has yet been reported, and ours is the first study to demonstrate that a quadratic equation for the K_a,r_ ratio can adequately explain the variation in the value of D_skin,max,RPLGD_/Total K_a,r_.

**Figure 6:**
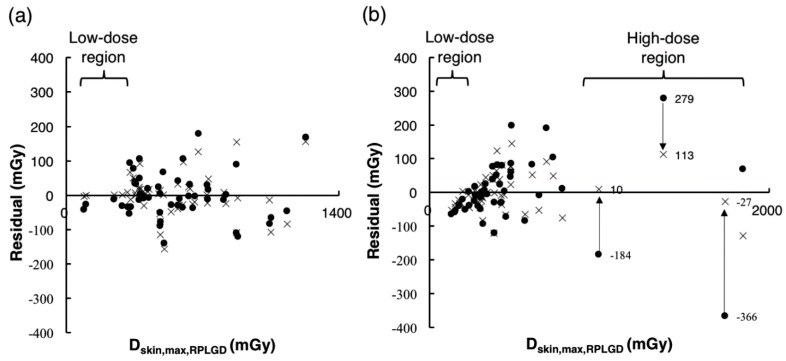
Residue plots of directly estimated D_skin,max RPLGD_ and indirectly estimated D_skin,max,Ka_. The residues of D_skin,max RPLGD_ − D_skin,max,Ka_ are plotted for (**a**) the test data set (*n* = 50) and (**b**) the validation data set (*n* = 50). The circles ● indicate the differences between the D_skin,max,Ka_ estimated using the CF_Ka,constant_ (Estimation Model 1) and D_skin,max RPLGD_, and the crosses × indicate the differences between D_skin,max,Ka_ estimated using the CF_Ka,function_ (Estimation Model 2) and D_skin,max RPLGD_. In the low-dose region of the test data set and the low-dose and high-dose regions of the validation data set, the residues with negative values when estimated using Estimation Model 1 were close to zero when Estimation Model 2 was used, highlighting the corrective effect of CF_Ka,function_.

Figure 6 shows the residue plots for directly estimated D_skin,max,RPLGD_ and indirectly estimated D_skin,max,Ka_. Applying the CF_Ka,function_ to the K_a,r_ ratio quadratic equations (see Equation (5) for the test data set and Equation (6) for the validation data set) and estimating the individual D_skin,max,Ka_ for each patient revealed a strong corrective effect in the high-dose region of the validation data set and a weak corrective effect in the low-dose regions of the test data set and the validation data set (Figure 6). This may be because radiation exposure is high in therapeutic NIR procedures, such as cerebral aneurysm coil embolization, in which the K_a,r_ ratio is high because fluoroscopy is conducted over long periods from the same X-ray entrance angle, and the D_skin,max,RPLGD_/Total K_a,r_ also increases (Figure 4d), and application of a high CF_Ka,function_ value can be used to correct D_skin,max,Ka_ appropriately. Conversely, the procedure that most commonly involves a low radiation dose is diagnostic cerebral angiography, a standard procedure in which most of the radiation dose comes from exposure at the same X-ray entrance angle (mainly via posterior–anterior and/or left–right projection), resulting in a low K_a,r_ ratio and increasing the D_skin,max,RPLGD_/Total K_a,r_ (Figure 2d and Figure 4d). As in the case of a high radiation dose, a high CF_Ka,function_ value can also be applied for appropriate correction of D_skin,max,Ka_. Applying CF_Ka,function_ weighted by the K_a,r_ ratio therefore facilitates more accurate estimation of D_skin,max,Ka_.

In this study, our objective was to construct a CF_Ka,function_ using the Fluoroscopy K_a,r_ and the Total K_a,r_ data recorded in the DICOM-RDSR, but as X-ray entrance angle data are also recorded for each fluoroscopy and exposure event, analysis of these data may also enable us to develop an index of the degree of variation in the X-ray entrance angle, potentially further increasing the accuracy of estimating D_skin,max,Ka_. As DICOM-RDSR is currently obligatory for all angiography systems both in Japan and overseas, it is a tool that is readily available in most institutions. Further studies should be conducted to explore other potential uses of DICOM-RDSR to reduce patient radiation exposure.

## 5. Conclusions

In this study, it was suggested that multiplying a conversion factor using the quadratic function for the ratio of Fluoroscopy K_a,r_/Total K_a,r_ for each patient by the Total K_a,r_ provides a more accurate estimate than multiplying with a constant conversion factor during cerebral angiography, including NIR procedures, in real time.

## Figures and Tables

**Table 1:** Example of a DICOM—Radiation Dose Structured Report (DICOM-RDSR) at our hospital.

Acquisition No.	No. of Frames	Fluoroscopy /Exposure	LAO /RAO (Degree)	CAUD /CRAN (Degree)	Tube Voltage (kV)	Tube Current (mA)	Pulse Width (ms)	SID (mm)	K_a,r_ (mGy)	Collimated Field Size (m^2^)	Pulse Rate (Pulse/s)	Fluoroscopy Time (s)
1	819	Fluoroscopy	LAO 0	CRAN 0	76	6.9	66.7	976	7.71	0.023	15	54.6
2	28	Fluoroscopy	RAO 30	CRAN 0	82	7.9	66.7	1041	0.34	0.020	15	1.9
3	11	Exposure	RAO 30	CRAN 0	71	654.0	41.4	1005	37.39	0.021	NA	−
4	195	Fluoroscopy	RAO 30	CRAN 0	82	7.9	66.7	1005	2.63	0.021	15	13.0
5	1938	Fluoroscopy	RAO 30	CRAN 0	82	7.9	66.7	1005	26.04	0.021	15	129.2
6	43	Fluoroscopy	LAO 0	CRAN 20	84	8.2	66.7	950	0.68	0.018	15	2.9
7	104	Fluoroscopy	LAO 0	CRAN 20	83	8.1	66.7	919	1.54	0.019	15	6.9
8	14	Fluoroscopy	LAO 0	CRAN 25	83	8.1	66.7	904	0.19	0.020	15	1.0
9	28	Exposure	LAO 0	CRAN 25	70	642.0	39.0	904	86.06	0.020	NA	−
10	329	Fluoroscopy	LAO 0	CRAN 0	78	7.2	66.7	957	3.56	0.018	15	21.9
∙	∙	∙	∙	∙	∙	∙	∙	∙	∙	∙	∙	∙
∙	∙	∙	∙	∙	∙	∙	∙	∙	∙	∙	∙	∙
∙	∙	∙	∙	∙	∙	∙	∙	∙	∙	∙	∙	∙
61	30	Fluoroscopy	LAO 0	CRAN 0	76	6.7	66.7	1016	3.02	0.021	15	2.0
62	767	Fluoroscopy	LAO 0	CRAN 0	81	7.8	66.7	1016	9.89	0.021	15	51.1

DICOM: Digital Imaging and Communication in Medicine; LAO: left anterior oblique; RAO: right anterior oblique; CAUD: caudal; CRAN: cranial; SID: source image receptor distance; K_a,r_: air kerma at the patient entrance reference point.

**Table 2:** Data set characteristics.

	Test Data Set	Validation Data Set	*p*-Value *
Period of investigation	October 2015–July 2016	August 2016–September 2017	
Number of cases (NIR)	50 (7)	50 (7)	
Men	20 (40%)	24 (48%)	
Women	30 (60%)	26 (52%)	
Age, y [range]	57.5 ± 13.9 [30–78]	62.8 ± 14.4 [33–88]	N.S.
BMI, kg·m^−2^ [range]	22.5 ± 3.1 [16.8–32.4]	−23.5 ± 3.8 [16.9–33.4]	N.S.
D_skin,max,RPLGD_, mGy	552.4 ± 250.3	457.9 ± 353.6	N.S.
Total K_a,r_, mGy	951.2 ± 398.2	807.8 ± 548.5	N.S.
Fluoroscopy K_a,r_, mGy	117.3 ± 151.8	124.5 ± 185.5	N.S.
Exposure K_a,r_, mGy	833.9 ± 322.7	683.3 ± 397.5	*p* = 0.04
Fluoroscopy Time, min	11.9 ± 10.1	12.7 ± 14.4	N.S.
Number of DSA	12.7 ± 5.4	11.3 ± 8.8	N.S.
Number of Frames	322.8 ± 100.5	297.8 ± 155.9	N.S.

All data are expressed as the mean ± standard deviation; * Welch’s *t*-test; N.S.: not significant; NIR: neurointerventional radiology; BMI: body mass index; D_skin,max,RPLGD_: the maximum absorbed dose to the most heavily irradiated localized region that was obtained using 64 radio-photoluminescence glass dosimeters placed on the surface of the head and neck of the patient (RADIREC^®^ system); K_a,r_: air kerma of the primary X-ray beam measured under specific conditions and expressed as the equivalent value at the patient entrance reference point; Total K_a,r_ = Fluoroscopy K_a,r_ + Exposure K_a,r_; DSA: digital subtraction angiography.

**Table 3:** Radio-photoluminescence glass dosimeter (RPLGD) energy compensation factors weighted by the ratio of the dose at the patient entrance reference point (K_a,r_ ratio) (test data set: *n* = 50).

Case No.	Fluoroscopy			Exposure			K_a,r_ Ratio		CF_RPLGD,weighted_
	Tube Voltage * (kV)	Effective Energy ** (keV)	CF_RPLGD_ *** (a)	Tube Voltage * (kV)	Effective Energy ** (keV)	CF_RPLGD_ *** (b)	Fluoroscopy K_a,r_/Total K_a,r_ (c)	Exposure K_a,r_/Total K_a,r_ (d)	
1	79.3	51.8	0.302	69.4	29.1	0.269	0.074	0.926	0.271
2	74.9	50.5	0.295	68.6	28.9	0.269	0.074	0.926	0.271
3	83.8	53.1	0.311	71.0	29.4	0.268	0.278	0.722	0.280
∙	∙	∙	∙	∙	∙	∙	∙	∙	∙
∙	∙	∙	∙	∙	∙	∙	∙	∙	∙
∙	∙	∙	∙	∙	∙	∙	∙	∙	∙
48	73.2	50.0	0.292	68.0	28.8	0.270	0.087	0.913	0.272
49	74.8	50.5	0.295	68.3	28.8	0.270	0.139	0.861	0.273
50	74.3	50.3	0.294	68.4	28.8	0.269	0.046	0.954	0.271
Mean	76.8	51.1	0.298	70.0	29.2	0.268	0.112	0.888	0.272
S.D.	3.4	1.0	0.006	2.0	0.4	0.001	0.091	0.091	0.004
Range	69.7—87.3	49.0—54.2	0.287—0.318	67.5—76.2	28.7—30.5	0.265—0.270	0.034—0.378	0.622—0.966	0.267—0.284

CF_RPLGD_: RPLGD energy compensation factor; RPLGD: radio-photoluminescence glass dosimeter; CF_RPLGD,weighted_: K_a,r_ ratio weighted RPLGD energy compensation factor obtained by the equation as follows: CF_RPLGD,weighted_ = (a) × (c) + (b) × (d); * Mean tube voltage for each patient derived from DICOM-RDSR; ** Effective energy value for the mean tube voltage for each patient. Calculated by interpolation from the NIST Standard Reference Database 126 [27] data; *** Calculated from the CF_RPLGD_ X-ray effective energy function (Equation (1)) shown in Figure 1.

**Table 4:** RPLGD energy compensation factors weighted by the K_a,r_ ratio (validation data set: *n* = 50).

Case No.	Fluoroscopy			Exposure			K_a,r_ Ratio		CF_RPLGD,weighted_
	Tube Voltage * (kV)	Effective Energy ** (keV)	CFRPLGD *** (a)	Tube Voltage * (kV)	Effective Energy ** (keV)	CFRPLGD *** (b)	Fluoroscopy K_a,r_/Total K_a,r_ (c)	Exposure K_a,r_/Total K_a,r_ (d)	
51	76.7	51.0	0.298	69.3	29.0	0.269	0.055	0.945	0.270
52	80.0	52.0	0.303	70.0	29.2	0.268	0.075	0.925	0.271
53	77.6	51.3	0.299	68.8	28.9	0.269	0.136	0.864	0.273
∙	∙	∙	∙	∙	∙	∙	∙	∙	∙
∙	∙	∙	∙	∙	∙	∙	∙	∙	∙
∙	∙	∙	∙	∙	∙	∙	∙	∙	∙
98	73.1	50.0	0.292	67.9	28.7	0.270	0.120	0.880	0.272
99	74.8	50.5	0.295	70.1	29.2	0.268	0.097	0.903	0.271
100	75.8	50.7	0.296	69.2	29.0	0.269	0.292	0.708	0.277
Mean	77.3	51.2	0.299	69.3	29.0	0.269	0.124	0.876	0.273
S.D.	2.9	0.8	0.005	1.5	0.3	0.001	0.090	0.090	0.004
Range	73.1—85.9	50.0—53.7	0.292—0.315	67.7—78.4	28.7—31.0	0.264—0.270	0.039—0.416	0.584—0.961	0.270—0.287

CF_RPLGD_: RPLGD energy compensation factor; CF_RPLGD,weighted_: K_a,r_ ratio weighted RPLGD energy compensation factor obtained by the equation as follows: CF_RPLGD,weighted_ = (a) × (c) + (b) × (d); * Mean tube voltage for each patient derived from DICOM-RDSR; ** Effective energy value for the mean tube voltage for each patient. Calculated by interpolation from the NIST Standard Reference Database 126 [27] data; *** Calculated from the CF_RPLGD_ X-ray effective energy function (Equation (1)) shown in Figure 1.
